# The Short-Term Effects of Visibility and Haze on Mortality in a Coastal City of China: A Time-Series Study

**DOI:** 10.3390/ijerph14111419

**Published:** 2017-11-20

**Authors:** Shaohua Gu, Jun Yang, Alistair Woodward, Mengmeng Li, Tianfeng He, Aihong Wang, Beibei Lu, Xiaobo Liu, Guozhang Xu, Qiyong Liu

**Affiliations:** 1 Ningbo Municipal Center for Disease Control and Prevention, Ningbo 315010, China; gushaohua1989@sina.com (S.G.); hetf@nbcdc.org.cn (T.H.); wangah@nbcdc.org.cn (A.W.); Lu1130@163.com (B.L.); 2 Institute for Environmental and Climate Research, Jinan University, Guangzhou 510000, China; smart_yjun@163.com; 3 School of Population Health, University of Auckland, Auckland 92019, New Zealand; a.woodward@auckland.ac.nz; 4 Department of Epidemiology and Biostatistics, Institute of Basic Medical Sciences, Chinese Academy of Medical Sciences, School of Basic Medicine, Peking Union Medical College, Beijing 100005, China; limm55@126.com; 5 State Key Laboratory of Infectious Disease Prevention and Control, Collaborative Innovation Center for Diagnosis and Treatment of Infectious Diseases, National Institute for Communicable Disease Control and Prevention, Chinese Center for Disease Control and Prevention, Beijing 102206, China; liuxiaobo@icdc.cn

**Keywords:** visibility, haze, ambient air pollution, mortality, vulnerable populations

## Abstract

Few studies have been conducted to investigate the acute health effects of visibility and haze, which may be regarded as proxy indicators of ambient air pollution. We used a distributed lag non-linear model (DLNM) combined with quasi-Poisson regression to estimate the relationship between visibility, haze and mortality in Ningbo, a coastal city of China. We found that the mortality risk of visibility was statistically significant only on the current day, while the risk of haze and PM_10_ peaked on the second day and could last for three days. When the visibility was less than 10 km, each 1 km decrease of visibility at lag 0 day was associated with a 0.78% (95% CI: 0.22–1.36%) increase in total mortality and a 1.61% (95% CI: 0.39–2.85%) increase in respiratory mortality. The excess risk of haze at lag 0–2 days on total mortality, cardiovascular and respiratory mortality was 7.76% (95% CI: 3.29–12.42%), 7.73% (95% CI: 0.12–15.92%) and 17.77% (95% CI: 7.64–28.86%), respectively. Greater effects of air pollution were observed during the cold season than in the warm season, and the elderly were at higher risk compared to youths. The effects of visibility and haze were attenuated by single pollutants. These findings suggest that visibility and haze could be used as surrogates of air quality where pollutant data are scarce, and strengthen the evidence to develop policy to control air pollution and protect vulnerable populations.

## 1. Introduction

Air pollution has become one of the biggest public health issues in the world. Increasing evidence has shown that short-term exposure to a single pollutant, such as particulate matter (PM) or gaseous pollutants, was significantly associated with various health outcomes, including increased cardiovascular and respiratory mortality, increased hospital visits for cardiac arrhythmia, asthma attacks, acute bronchitis and chronic obstructive pulmonary disease (COPD) and decreased lung function [1,2,3,4,5,6,7]. According to the global burden of disease in 2015, a total of 4.2 million deaths were attributed to ambient fine particle air pollution (PM_2.5_) and 59% of these deaths occurred in east and south Asia [8]. An effective air pollution warning system is warranted to alert the public, communities, and relevant authorities and help to allocate the public health and social resources to combat the adverse effects of air pollution, which ultimately could save human lives. However, since air pollution monitoring data are insufficient in many developing countries, air pollution warning systems are not well established in these areas [9]. Therefore, it is important to detect a proxy indicator of air quality which is commonly available in these countries.

Visibility is usually regarded as a primary index of air quality and can be easily measured at airport and meteorological stations [10,11]. The concentration of fine particulate matter is the dominant factor that determines the change of visual range [12,13]. Its major components, such as sulfate, nitrate and organic matter can reduce visibility by absorbing and scattering the ambient light [14]. As a special condition of visibility, haze may provide a better indicator of air quality due to the removal of the confounding effects of other weather conditions such as fog, rain and snow [13,15]. However, as surrogates for air pollution, there were very limited numbers of studies assessing the health burdens of visibility and haze [10,11,15]. In addition, all of the above-mentioned studies assessed the relationships between health outcomes and visibility and haze separately, and did not involve a comparison between the effect patterns of different indicators simultaneously.

With the rapid development of the social economy, ambient air pollution has become serious public health problem in China, especially in urban areas such as the Beijing–Tianjin region, Yangtze River Delta (YRD) and Pearl River Delta (PRD) [16,17,18]. Dust haze, as the air pollution that people can see, has attracted increasing concern from the public and local governments in recent years. Although a nationwide regulatory air pollution monitoring network has been built since the end of 2012 in China, some heavily polluted areas are still not covered [16]. In order to better understand the effect of air pollution, we used a time-series study to estimate the relationship between visibility, haze and mortality in Ningbo city and to explore the effects of seasonal variation and vulnerable populations. 

## 2. Materials and Methods

### 2.1. Study Site

The Ethics Committee of Chinese Center for Disease Control and Prevention where this study was conducted approved the study proposal (No. 201214). As one of the central cities in the YRD, Ningbo had less than 15 haze days per year before 2000, but nearly 50 haze days per year after 2000 [13]. The city has a humid subtropical monsoon climate and four distinctive seasons. In the end of 2013, the number of registered residents of Ningbo was 5.8 million, distributed in an area of approximately 9.816 square kilometers [4]. In recent years, Ningbo frequently experienced serious pollution accidents, such as long periods of haze events in the winter of 2013, during which the daily maximum concentration of PM_10_ was more than 600 µg/m^3^.

### 2.2. Data Collection

The daily counts of death data were obtained from Ningbo Municipal Center for Disease Control and Prevention (Ningbo CDC) from 1 January 2011 to 31 December 2013. The data were quality checked by the Ningbo CDC and those who died in Ningbo but lived temporarily (non-registered residents) were excluded [4]. The causes of death were coded according to the tenth revision of the International Statistical Classification of Disease (ICD-10), and the codes A00-Z99, I00-I99 and J00-J99 represent total mortality, cardiovascular disease (CVD) and respiratory disease (RD), respectively. In addition, the data on total mortality were further classified by individual characteristics, including gender and age (0–64 and 65+ years).

Daily concentrations of PM_10_, NO_2_ and SO_2_ were collected from the Environment Monitoring Center of Ningbo. Daily meteorological data, including horizontal visibility, daily precipitation, average temperature, relative humidity, air pressure and wind speed were collected from the China Meteorological Data Sharing Service System. All environmental factors were measured at Yinzhou station, which was located in the center of Ningbo city. Visibility was observed by professional meteorologists four times (2:00, 8:00, 14:00, 20:00) per day, and these records were averaged to provide daily average visibility [13]. According to the definition from the China Meteorological Administration and previous studies, a haze event was defined as daily average visibility <10 km and relative humidity <80% on non-precipitation day [13,19,20].

### 2.3. Statistical Analysis

Time series analysis has been commonly used to study the short-term effects of air pollution, and its feature is that the likelihood of confounding effects of outcome variables is minimized by other factors [10,21]. We used a distributed lag non-linear model (DLNM) combined with quasi-Poisson regression to estimate the effects of visibility and haze on mortality, adjusting for potential confounding factors, such as long-term and seasonal trends, relative humidity and air pressure [22,23,24]. As the representative indicator of air pollution, PM_10_ was also used to examine effects on mortality in the same way. The Akaike information criterion for quasi-Poisson (Q-AIC) was applied to verify the optimal degree of freedom (df) of models [21,22,23]. The model can be expressed as follows:

(1) $$log\left[E\left(Y_{t}\right)\right]=\alpha +ns\left(Time_{t},7\times 3\right)+ns\left(TEMP_{t},6\right)+ns\left(RH_{t},3\right)+ns\left(PRESS_{t},3\right)+\upsilon Dow_{t}+\eta Holiday_{t}+\beta AP_{t,l}$$

where Y*_t_* is the number of observed deaths at calendar day *t* (1, 2, …, 1096); α is the intercept; *ns*() means a natural cubic spline; 7 df per year for time was used to control for the long-term trend and seasonality of daily mortality [22,24,25]; 6 df was used for average temperature (*TEMP_t_*), which was consistent with a previous study [6], and 3 df for relative humility (*RH_t_*) and atmospheric pressure (*PRESS_t_*), respectively [4,21]. Day of the week (*Dow_t_*) and public holidays (*Holiday_t_*) were also included in the model as indicator variables, and υ and η were the corresponding vector of coefficients; *AP_t,l_* represented the cross-basis matrix produced by DLNM to fit the distributed lag effects of visibility, haze and PM_10_; to make it compare to the effect of haze, we used the linear threshold model to analysis the effect of visibility, using the same threshold of 10 km; haze was a binary variable (0-non-haze day and 1-haze day), and “non-haze day” was used as the reference group [15]. Given a consistently linear association between PM_10_ concentration and mortality [4,21,26], the linear function was used to analyze the effects of PM_10_ on mortality; the maximum lag days of air pollution was set as seven days, and the dfs for the lag structures were chosen based on Q-AIC; β is vector of coefficients for *AP_t,l_*. Similar methods were also used for the cold season (from November to April) and warm season (from May to October), but the df of time trend was changed to three per year. The excess risk (ER%) was used to express the percent increase in daily mortality with a unit change of air pollution and it was calculated as 100% × (e*^β^*−1).

A series of sensitivity analyses were performed to test the robustness of our results by changing the df for the time trend from six to eight per year, the maximum lag days of haze events from three to ten, and other parameters. A different haze definition, daily average visibility <10 km and relative humidity <90% on a non-precipitation day, was also performed [13,15].

All analyses were performed using the R software (vision 3.1.0; R Development Core Team 2010), and the “dlnm” package was used to fit the DLNM model. All statistical tests were two-sided and *p* < 0.05 was considered statistically significant.

## 3. Results

**Figure 1:**
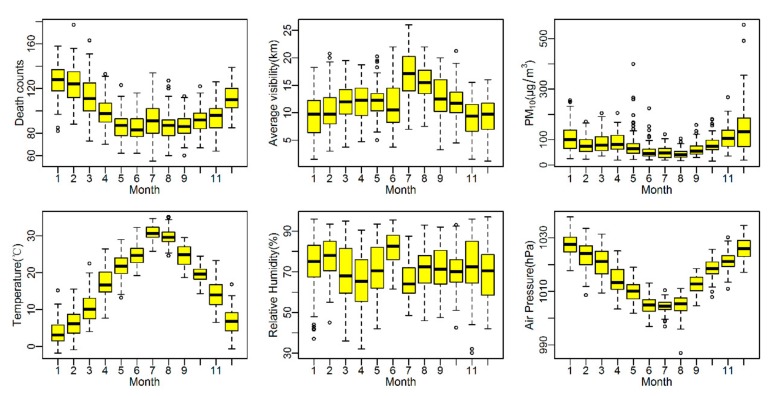
Boxplots of monthly death counts, PM_10_ concentration, temperature, relative humidity, air pressure and average visibility in Ningbo, China, 2011–2013. Boxplots of monthly death counts, PM_10_ concentration, temperature, relative humidity, air pressure and average visibility in Ningbo, China, 2011–2013.

Table 1 shows the descriptive statistics of dependent and independent variables. A total of 109,210 deaths were collected from 2011 to 2013 in Ningbo city, while the number of cardiovascular and respiratory mortalities was 30,258 and 17,571, accounting for 27.71% and 16.09% of total deaths, respectively. Daily average visibility was 11.9 ± 4.2 km, and there were 96 (8.76%) haze days identified during the study period, most of which occurred in the cold season (October to April). Figure 1 shows the boxplots of daily deaths counts, meteorological factors and air pollution during 2011–2013. Visibility showed a seasonal trend with lower values in the cold season, and vice versa for death counts and PM_10_ concentration.

The Spearman’s correlations between daily visibility and single pollutants were all statistically significant (*p* < 0.05), and the corresponding coefficients were higher for NO_2_ (*r*_s_ = −0.477) and relatively lower for PM_10_ (*r*_s_ = −0.415) and SO_2_ (*r*_s_ = −0.355). After excluding non-haze days, the correlation coefficients were higher for PM_10_ (*r*_s_ = −0.624), followed by NO_2_ (*r*_s_ = −0.477) and SO_2_ (*r*_s_ = −0.115). During haze days, the average concentration of PM_10_, SO_2_, NO_2_ were 159 μg/m^3^, 40 μg/m^3^ and 66 μg/m^3^, all of which were significantly greater than those corresponding values (75 μg/m^3^, 21 μg/m^3^ and 44 μg/m^3^) on non-haze days. Additionally, the visibility, temperature and relative humidity were much lower during the haze days, but with higher atmospheric pressure.

**Figure 2:**
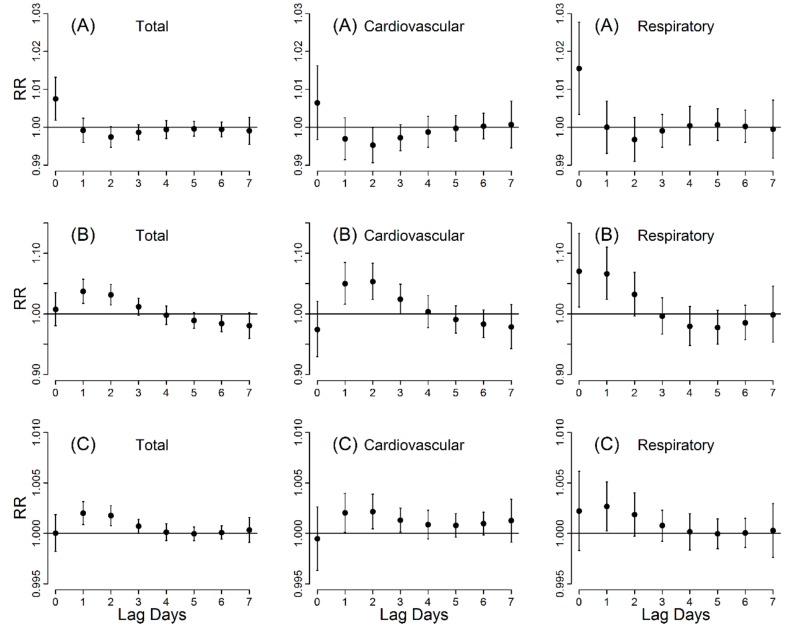
Relative risk (RR, 95% CI) for mortality with per 1 km decrease of visibility (**A**), in haze days (**B**) and per 10 µg/m^3^ increase of PM_10_ concentration (**C**) at various lag days. The threshold of visibility was 10 km.

Figure 2 shows the lag patterns of visibility, haze and PM_10_ on mortality. Generally, after exposure to air pollution, the mortality risk of visibility was statistically significant only on the current day, while the risk of haze and PM_10_ peaked on the second day and could last for three days. When the visibility less than 10 km, each 1 km decrease of visibility at lag day 0 were associated with a 0.78% (95% CI: 0.22–1.36%) increase in total mortality, a 0.71% (95% CI: −0.27–1.70%) increase in cardiovascular mortality, and a 1.61% (95% CI: 0.39–2.85%) increase in respiratory mortality. The excess risk of haze at 0–2 lag days on total mortality, cardiovascular and respiratory mortality was 7.76% (95% CI: 3.29–12.42%), 7.73% (95% CI: 0.12–15.92%) and 17.77% (95% CI: 7.64–28.86%), respectively.

Table 2 presents the excess risk of air pollution on mortality by season and individual factors. The effects of visibility, haze and PM_10_ were greatly enhanced during the cold season, while statistically insignificant effects were found in the warm season. The elderly were consistently at higher risk of impaired visibility, haze and PM_10_. Males were at higher risk of impaired visibility, while females were at higher risk for haze and PM_10_.

Table 3 shows the effects of visibility and haze on mortality, with adjustment for different pollutants. After adjustment for PM_10_, SO_2_ and NO_2_ as confounding variables, the effects of visibility and haze were attenuated. However, the effect estimates were only halved and became non-significant even though all the three pollutants were controlled for. The results from the sensitivity analysis indicated that the effects of visibility and haze on total mortality were robust after changing the df for time trends, the maximum lag days and other parameters, and the effect estimates slightly decreased when haze was defined as a cut-off limit for humidity less than 90% (Table 4).

## 4. Discussion

This study found that visibility and haze were significantly associated with an increased risk of mortality in Ningbo city, with a higher impact on respiratory mortality and the elderly. The effects of visibility and haze were strongly correlated with PM_10_, NO_2_ and SO_2_, but could not be totally explained by these single air pollutants. The results suggest that visibility and haze can be used as proxy indictors of air quality, and provide a useful understanding of the short-term effect of air pollution on mortality in China.

Impaired visibility is often called “the pollution people see”, and is mainly caused by an increase in particulate matter, photochemical smog and other environmental factors [12]. Consistent with the study of Liu et al. [15], we found that haze had very large effects on mortality compared with visibility, and the effects were attenuated after adjusting for single pollutants (PM_10_, NO_2_ and SO_2_). PM is regarded as the major contributor to impaired visibility and haze. After removing confounding effects, the lag patterns of haze and PM_10_ on mortality were very similar. In addition to PM_10_, NO_2_ and SO_2_ may also contribute to visibility and haze events. However, our study found that the effects of visibility and haze could not be totally explained by PM_10_, NO_2_ and SO_2_, demonstrating that these phenomena are composed of multiple chemical substances, including other sources, such as volatile organic compounds, organic carbon, O_3_ and PM_2.5_. Therefore, it is important to quantify the health burden of visibility and haze from the public health point of view, since they are the most familiar phenomenon of air pollution for the public and local governments in China. Furthermore, given the interaction or confounding effect between different pollutants, it is difficult to estimate the integrated effect of ambient air pollution on health [27,28]. Our findings suggest that the study of visibility and haze could provide a better understanding of the integrated effect of air pollution. 

We also found that the risk and lag structure of haze effect varied by different causes of death. In this study, the maximum risk of cardiovascular and respiratory mortality was found on the third day (lag2) and current day (lag0) after exposure to haze, respectively. Previous investigations also confirmed that the short-term effect of haze on respiratory mortality is stronger and more acute, which may be explained by the biological mechanism for the adverse health impact of air pollution [11,15]. As the primary component of haze, PM can be inhaled into the lungs and directly damage lung tissues through inflammatory effects, cytokine production and the enhancement of allergic responses in the upper and lower airways [29]. On the other hand, PM is also a cardiovascular risk factor which can cause systemic inflammation, systemic oxidative stress, altered autonomic regulation of heart rate and changes in the clotting abilities of the blood [30]. 

The study shows that season, age and gender may modify the association between air pollution and human health, confirming that the effect of air pollution was more significant during the cold season, and that the elderly were the vulnerable subpopulation in Ningbo city. Reduced physiological function, passive indoor/outdoor activity, poor social networks and existing chronic health problem (e.g., cardiorespiratory disease and diabetes) may contribute to the elderly’s vulnerability to air pollution [26,31,32,33]. However, the modification by gender varied by location and study [6,15,26,31]. A systematic review studied 36 papers from the whole world and concluded that there is weak evidence that particulate matter exposure risks are higher for women than for men [33]. Gender susceptibility to air pollution may be related to differences in hormonal changes, lung function, dermal absorption, physical activity and personal care products [4,26]. The study on modifiers for risks indicates that vulnerable people should be more concerned when developing policy for air pollution control.

As the most familiar phenomenon of air pollution for the public and local governments, the study may help them to better understand the adverse health impacts of visibility and haze, and provide a unique evidentiary basis for policy-making on air pollution control in China. Some limitations should also be considered in this study. Firstly, only one visibility monitoring station was found in Ningbo city, and its data may not be sufficient to represent the whole region. This may cause a limited influence on the effect estimate but hardly change the trend. Secondly, since visibility can be influenced by other weather phenomena, such as fog and rain, haze may be misclassified due to the cut-off limit of humidity. However, in the sensitivity analysis, we changed the definition of haze with a cut-off limit for humidity less than 90%, and the impact was still robust. Finally, as with other time-series studies, data on air pollution and mortality were collect at the population level rather than the individual level. Thus, we could not exclude the possibility of ecological bias and caution should be exercised in drawing a casual association between the underlying effect of visibility and mortality [11,15].

## 5. Conclusions

This study suggests that short-term exposure to visibility and haze were associated with mortality risk, especially for the elderly. From a public health view, our findings suggest that visibility and haze could be used as suitable surrogates for air quality where air pollution data are scarce, and provide effective evidence to strengthen public awareness of potentially harmful effects of ambient air pollution. More relevant policies should be applied to reduce the health impact of air pollution, especially for those vulnerable subpopulations.

## Figures and Tables

**Table 1:** Summary statistics for daily death counts, meteorological factors and air pollution in Ningbo city, during 2011–2013.

Variables	Mean ± SD	Minimum	Percentiles			Maximum
			25th	50th	75th	
Daily death counts						
Total	99.6 ± 19.9	55	85	96	112	177
Cardiovascular	27.6 ± 8.3	8	22	27	33	60
Respiratory	16.0 ± 6.6	2	11	15	20	45
Gender						
Male	56.0 ± 11.4	27	48	55	63	96
Female	43.7 ± 11.0	19	35	42	51	85
Age (years)						
<65	24.1 ± 5.3	10	20	24	28	41
≥65	75.6 ± 18.0	35	62	72	87	143
Meteorological variables						
Visibility (km)	11.9 ± 4.2	1.2	9.0	11.8	14.5	26.0
Temperature (°C)	17.6 ± 9.4	−1.8	9.3	18.9	25.3	35.1
Relative humidity (%)	71.7 ± 12.7	30.0	63.0	71.8	81.0	97.0
Atmosphere pressure (hPa)	1015.7 ± 9.1	987.0	1007.6	1016.1	1022.7	1037.8
Air pollution						
PM_10_ (μg/m^3^)	82.1 ± 53.4	15	47	69	104	554
SO_2_ (μg/m^3^)	22.5 ± 17.6	2	10	17	31	131
NO_2_ (μg/m^3^)	45.8 ± 22.7	1	29	44	61	154

**Table 2:** The excess risk (ER%, 95% CI) for daily mortality with per 1 km decrease of visibility at lag day 0, per 10 µg/m^3^ increase of PM_10_ concentration and in haze days during 0–2 lag days, stratified by season and individual groups.

Variables	Visibility			Haze			PM_10_		
	Warm Season	Cold Season	Full Year	Warm Season	Cold Season	Full Year	Warm Season	Cold Season	Full Year
Mortality									
Total	−0.59 (−1.79–0.63)	**1.18** **(0.50–1.86)**	**0.78** **(0.22–1.36)**	6.41 (−3.61–17.47)	**10.01** **(5.03–15.40)**	**7.76** **(3.29–12.42)**	0.47 (−0.09–1.02)	**0.43** **(0.18–0.68)**	**0.38** **(0.17–0.59)**
Cardiovascular	−1.91 (−3.97–0.20)	**1.30** **(0.11–2.50)**	0.71 (−0.27–1.70)	6.11 (−10.70–26.08)	**12.08** **(3.29–21.62)**	**7.73** **(0.12–15.92)**	0.25 (−0.72–1.23)	**0.49** **(0.07–0.92)**	**0.37** **(0.01–0.73)**
Respiratory	1.63 (−1.24–4.58)	**1.53** **(0.10–2.98)**	**1.61** **(0.39–2.85)**	12.40 (−11.16–42.22)	**23.70** **(12.24–36.32)**	**17.77** **(7.64–28.86)**	0.67 (−0.65–2.01)	**0.82** **(0.31–1.33)**	**0.68** **(0.22–1.13)**
Gender									
Male	0.12 (−1.41–1.67)	**1.30** **(0.45–2.16)**	**1.01** **(0.29–1.74)**	4.73 (−7.64–18.75)	**9.87** **(3.56–16.56)**	**6.88** **(1.27–12.79)**	0.58 (−0.12–1.29)	0.29 (−0.02–0.60)	**0.33** **(0.06–0.60)**
Female	−1.52 (−3.29–0.29)	**1.03** **(0.11–1.96)**	0.50 (−0.30–1.30)	8.64 (−6.37–26.06)	**10.47** **(3.68–17.71)**	**8.93** **(2.62–15.64)**	0.32 (−0.51–1.14)	**0.60** **(0.27–0.93)**	**0.44** **(0.15–0.74)**
Age (years)									
<65	−0.90 (−3.05–1.30)	1.00 (−0.24–2.26)	0.60 (−0.44–1.64)	−3.07 (−19.05–16.08)	1.84 (−6.66–11.12)	0.24 (−7.34–8.44)	0.34 (−0.64–1.33)	0.03 (−0.42–0.49)	0.11 (−0.28–0.50)
≥65	−0.48 (−1.85–0.91)	**1.23** **(0.45–2.01)**	**0.84** **(0.19–1.49)**	10.09 (−1.70–23.29)	**12.61** **(6.80–18.73)**	**10.09** **(4.94–15.49)**	0.51 (−0.12–1.15)	**0.54** **(0.26–0.82)**	**0.46** **(0.22–0.70)**

Note: Statistically significant (*p* < 0.05) were labeled in bold font. The threshold of visibility was 10 km.

**Table 3:** Excess risk (ER%) for mortality with per 1 km decrease of visibility at lag day 0 and in haze days at 0–2 lag days, after adjustment for different pollutants.

Air Pollution and Model	Total		Cardiovascular		Respiratory	
	ER (%)	95% CI	ER (%)	95% CI	ER (%)	95% CI
Visibility						
Single model	**0.78**	**0.22–1.36**	0.71	−0.27–1.70	**1.61**	**0.39–2.85**
+PM_10_	**0.70**	**0.05–1.35**	0.83	−0.29–1.97	**1.03**	**0.36–2.45**
+SO_2_	**0.67**	**0.07–1.28**	0.59	−0.45–1.63	1.22	−0.09–2.54
+NO_2_	**0.69**	**0.09–1.29**	0.61	−0.42–1.66	**1.42**	**0.12–2.74**
+PM_10_ + SO_2_ + NO_2_	**0.71**	**0.04–1.38**	0.90	−0.26–2.06	0.87	−0.57–2.33
Haze						
Single model	**7.76**	**3.29–12.42**	**7.73**	**0.12–15.92**	**17.77**	**7.64–28.86**
+PM_10_	**5.44**	**0.34–10.79**	6.04	−2.67–15.53	**13.85**	**2.44–26.53**
+SO_2_	**5.43**	**0.90–10.17**	3.16	−4.39–11.31	**14.05**	**3.78–25.34**
+NO_2_	**6.09**	**1.50–10.89**	4.96	−2.76–13.31	**15.76**	**5.29–27.27**
+PM_10_ + SO_2_ + NO_2_	**5.59**	**0.35–11.12**	5.61	−3.27–15.31	**13.11**	**1.36–26.23**

Note: Statistically significant (*p* < 0.05) were labeled in bold font. The threshold of visibility was 10 km.

**Table 4:** Sensitivity analyses of the excess risk (ER%) for total mortality with per 1 km decrease of visibility at lag day 0 and in haze days at 0–2 lag days, by changing maximum lag days, degree of freedom (df) and definition of haze.

Variables	Visibility		Haze	
	ER (%)	95% CI	ER (%)	95% CI
1. Maximum lag days				
3	**0.79**	**0.22–1.37**	**8.98**	**4.33–13.85**
7	**0.78**	**0.22–1.36**	**7.76**	**3.29–12.42**
10	**0.78**	**0.23–1.34**	**6.79**	**2.37–11.39**
2. df for time				
6	**0.74**	**0.17–1.31**	**8.29**	**3.80–12.98**
7	**0.78**	**0.22–1.36**	**7.76**	**3.29–12.42**
8	**0.73**	**0.17–1.30**	**7.77**	**3.30–12.43**
3. df for temperature				
3	**0.80**	**0.23–1.37**	**7.62**	**3.13–2.31**
6	**0.78**	**0.22–1.36**	**7.76**	**3.29–12.42**
4. df for air pressure and relative humidity				
3	**0.78**	**0.22–1.36**	**7.76**	**3.29–12.42**
6	**0.70**	**0.12–1.27**	**7.37**	**2.88–12.06**
5. Definition of haze day				
Humidity < 80% *	-	-	**7.76**	**3.29–12.42**
Humidity <90%^#^	-	-	**6.45**	**2.42–10.64**

Note: Statistically significant (*p* < 0.05) were labeled in bold font. The threshold of visibility was 10 km. Haze was defined as daily average visibility <10 km and relative humidity <80% on a non-precipitation day; Haze was defined as daily average visibility <10 km and relative humidity <90% on a non-precipitation day.
